# EARLY NEONATAL NEAR MISS IN A UNIVERSITY HOSPITAL: COMPARATIVE CROSS-SECTIONAL STUDY

**DOI:** 10.1590/1984-0462/2021/39/2019317

**Published:** 2020-09-25

**Authors:** Karla Eveline Ximenes de França, Mirella Bezerra Rodrigues Vilela, Paulo Germano de Frias, Silvia Wanick Sarinho

**Affiliations:** a Universidade Federal de Pernambuco, Recife, PE, Brazil.; b Instituto de Medicina Integral Professor Fernando Figueira, Recife, PE, Brazil.

**Keywords:** Healthcare near miss, Infant, newborn, Early neonatal mortality, Vital statistics, Information systems, Near miss, Recém-nascido, Mortalidade neonatal precoce, Estatísticas vitais, Sistemas de informação

## Abstract

**Objective:**

To compare 2012 and 2016 data on early neonatal near miss indicators from Health Information Systems at a university hospital.

**Methods:**

This is a cross-sectional study conducted in 2012 and 2016. We considered early neonatal near misses the live births that presented one of the following risk conditions at birth: gestational age <33 weeks, birth weight <1,750g or 5-minute Apgar score <7, or Neonatal Intensive Care Unit (NICU) admission, and were alive until the 7^th^ day of life. Data were collected from the Live Birth Information System, Hospital Information System, and Mortality Information System. We calculated the early neonatal mortality rate, neonatal near miss rate, severe neonatal outcome rate, early neonatal survival index, and early neonatal mortality index, compared by year of birth.

**Results:**

In 2012, 304 early neonatal near misses were registered, with a higher proportion of cases with very low birth weight and mothers who had zero to three prenatal visits. In 2016, the number of cases was 243, with a predominance of more NICU admissions. The incidence of early neonatal deaths and early neonatal near misses was higher in 2012 than in 2016.

**Conclusions:**

Neonatal near miss indicators identified difference between years. The cases were more severe in 2012 and there were more NICU admissions in 2016.

## RESUMO

**Objetivo:**

Comparar os anos de 2012 e 2016 quanto a indicadores de *near miss* neonatal precoce, com base nos dados de Sistemas de Informação em Saúde, em hospital universitário.

**Métodos:**

Estudo transversal realizado em 2012 e 2016. Consideraram-se casos de *near miss* neonatal precoce os nascidos vivos que apresentaram uma das condições de risco ao nascer (idade gestacional <33 semanas, peso ao nascer <1750 g, índice de Apgar no 5º minuto de vida <7, ou internação em unidade de terapia intensiva neonatal - UTIN) e que permaneceram vivos até o 7º dia de vida. Os dados foram obtidos do Sistema de Informações de Nascidos Vivos, do Sistema de Informações Hospitalares e do Sistema de Informação sobre Mortalidade. Calcularam-se a taxa de mortalidade neonatal precoce, a taxa de *near miss* neonatal, a taxa de desfecho neonatal grave, o índice de sobrevivência neonatal precoce e o índice de mortalidade neonatal precoce, comparados segundo o ano de nascimento.

**Resultados:**

Em 2012, ocorreram 304 casos de *near miss* neonatal precoce e maior proporção de casos com muito baixo peso ao nascer e de mães que realizaram nenhuma a três consultas de pré-natal. Em 2016 aconteceram 243 casos, com predomínio das internações em UTIN. O ano de 2012 teve mais óbitos neonatais precoces e mais casos de *near miss* neonatal precoce do que o de 2016.

**Conclusões:**

Os indicadores de *near miss* neonatal precoce identificaram diferenças entre os anos comparados, com maior gravidade em 2012 e, em contrapartida, maior número de internações em UTIN em 2016.

## INTRODUCTION

Despite the reduction in infant mortality that has occurred in Brazil in recent decades, neonatal mortality remains a public health problem.^1^ ^,^ ^2^ Neonatal deaths are related to the quality of health care provided for women and newborns since the prenatal period, and interventions aimed at this population group are necessary for the survival of severe cases.^3^ ^,^ ^4^ Studies on institutional neonatal mortality and on survivors of risk conditions at birth are regarded as instruments that reveal barriers to improving care.^5^ ^,^ ^6^

Neonatal near misses are newborns who almost died from severe complications in the first days of life but survived the neonatal period.^7^ ^,^ ^8^ They generally represent from three to ten times the number of neonatal deaths.^9^ ^,^ ^10^ ^,^ ^11^

Operational definitions of neonatal near miss, although not consensual,^5^ ^,^ ^8^ are generally based on pragmatic criteria: birth weight, gestational age, and 5-minute Apgar score.^7^ ^,^ ^11^ ^,^ ^12^ Other definitions are associated with the management variables used to save the baby’s life, such as blood transfusion, surfactant use, phototherapy, mechanical ventilation, etc.^13^ ^,^ ^14^

The use of neonatal near miss definitions to monitor care outcomes in health facilities is a challenge, but it can be easier with the variables available in the information systems maintained in daily services.^11^ ^,^ ^12^

Neonatal near miss indicators are used for diagnosis, monitoring, and evaluation of neonatal hospital care and make it possible to compare the same or different health facilities over time.^7^ ^,^ ^9^ The surveillance of neonatal near misses and the monitoring of their indicators may reveal weaknesses in health care and favor the promotion of public policies aimed at women, pregnant women, and newborns.^11^

Thus, the study aimed to compare 2012 and 2016 data on early neonatal near miss indicators from Health Information Systems in a university hospital.

## METHOD

This is a cross-sectional study performed at the Hospital Geral das Clínicas (HC) of the Universidade Federal de Pernambuco, a federal agency that provides services exclusively to the Brazilian public health system (*Sistema Único de Saúde* - SUS), located in the city of Recife, capital of Pernambuco, and which offers nursing, nutrition, and multidisciplinary residency programs. The institution has 15 beds for clinical obstetrics, 15 for surgery, 5 for the conventional neonatal intermediate care unit, and 10 for the neonatal intensive care unit (NICU). It performs approximately 130 deliveries per month and is a reference for high-risk pregnancy and delivery.^15^

We considered early neonatal near misses the live births that presented any of the following risk conditions at birth: gestational age <33 weeks, birth weight <1,750 g, 5-minute Apgar score <7, or NICU admission, and were alive until the 7^th^ day of life.^11^

Data from 2012 and 2016 were collected from the State Health Department: those related to live births were obtained from the Live Birth Information System (*Sistema de Informações de Nascidos Vivos* - Sinasc) and to early neonatal deaths, from the Mortality Information System (*Sistema de Informação sobre Mortalidade* - SIM). We used data from the SUS Hospital Information System (*Sistema de Informações Hospitalares do SUS* - SIH-SUS) to obtain the information on the NICU admission criteria, through the analysis of the hospital admission authorization copy of each hospitalized newborn.

**Figure 1:**
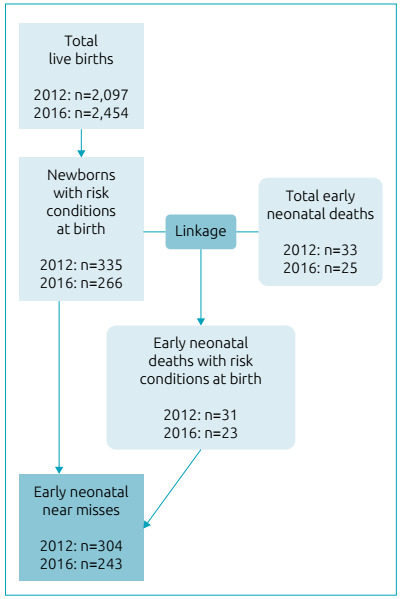
Flowchart of data processing. Hospital das Clínicas, Recife, Pernambuco, Brazil, 2012 and 2016.

We identified early neonatal near misses in 2016 by initially searching Sinasc for live births that presented the studied risk conditions at birth. As for the NICU admission criteria, these newborns were identified using SIH-SUS and subsequently located in the Sinasc database. Next, a deterministic linkage was carried out between the SIM, which included early neonatal deaths, and Sinasc databases, using the number of the live birth certificate found in the death certificate as the search field. A nominal search was performed using the mother’s name for the cases not paired in the previous step, and the confirmation of true pairs was obtained by the child’s sex and date of birth. Through the linkage, we identified early neonatal deaths of newborns who presented risk conditions at birth. Lastly, these cases were excluded from the sample so that only survivors remained, that is, early neonatal near misses (Figure 1). Information about early neonatal near misses that occurred in 2012 was extracted from a previous study.^11^

Early neonatal near misses were characterized based on maternal (maternal age; type of pregnancy; parity; number of prenatal visits) and newborn (sex; type of delivery; duration of pregnancy; birth weight; 5-minute Apgar score; NICU admission) variables, which were compared according to the year of birth using Pearson’s chi-square test, with α=5%.

The cases were also categorized by entry criteria to identify those that most contributed to classifying newborns as near misses and compared using Pearson’s chi-square test. The following neonatal near miss indicators were calculated:

- Early neonatal mortality rate (ENMR): number of early neonatal deaths divided by the total number of live births multiplied by 1,000.
- Neonatal near miss rate (NNMR): number of neonatal near misses divided by the total number of live births multiplied by 1,000.
- Severe neonatal outcome rate (SNOR): number of neonatal near misses added to early neonatal deaths divided by the total number of live births multiplied by 1,000.
- Early neonatal survival index (ENSI), suggested by this study: number of newborns surviving the first week of life among those with life-threatening conditions at birth divided by the total number of newborns with life-threatening conditions at birth multiplied by 100.
- Early neonatal mortality index (ENMI): number of newborn deaths in the first week of life among those with life-threatening conditions at birth divided by the total number of newborns with life-threatening conditions at birth multiplied by 100.

We used prevalence ratio to compare the indicators.

Death certificates and live births certificates with filling issues had the missing variables provided by a search in the hospital medical records and by the municipality’s Health Department, supported by the hospital epidemiology center. The same procedure was not followed for hospital admission authorizations.

Data collection, processing, and analysis took place from July 2018 to February 2019, using Microsoft Excel 2010 (Microsoft Corp., United States) and Epi-Info, version 7.1.5.2 (Centers for Disease Control and Prevention, Atlanta, United States).

 The Research Ethics Committee approved this study, under opinion numbers 1,226,298 (September 14, 2015) and 2,773,429 (July 17, 2018) and Certificate of Presentation for Ethical Consideration (*Certificado de Apresentação para Apreciação Ética* - CAAE) 47358315.1. 0000.5208 and 90684418.8.0000.5208.

## RESULTS

We identified 2,097 live births in 2012 and 2,454 in 2016 at the studied hospital. Among them, 304 were classified as early neonatal near misses in 2012 and 243 in 2016, respectively representing 9.21 and 9.72 times the number of early neonatal deaths.

Statistically significant differences were found regarding the type of pregnancy, with more early neonatal near misses resulting from twin pregnancies in 2016, and the number of prenatal visits, with a higher proportion of mothers who had zero to three visits in 2012. In addition, we found significant differences as to birth weight in 2012, with more than twice the proportion of early neonatal near misses presenting very low birth weight, and the need for NICU admission, which was higher in 2016 (Table 1).

**Table 1:** Maternal, biological, and birth variables of early neonatal near misses according to the year of birth. Hospital das Clínicas, 2012 and 2016.

	2012 (n=304)		2016 (n=243)		Total (n=547)		p-value^a^
	n	%	n	%	n	%	
Maternal age (years)							
10-19	88	28.9	66	27.2	154	28.1	0.85
20-35	188	61.8	152	62.6	340	62.2	
36 or older	28	9.2	25	10.3	53	9.7	
Type of pregnancy^b^							
Single	284	93.7	215	88.5	499	91.4	0.04
Multiple	19	6.3	28	11.5	47	8.6	
Parity							
1^st^ child	145	47.7	109	44.9	254	46.4	0.56
2^nd^ child or more	159	52.3	134	55.1	293	53.6	
Prenatal visits^c^							
0-3	66	22.2	36	14.9	102	18.9	0.01
4-6	132	44.4	98	40.5	230	42.7	
7 or more	99	33.3	108	44.6	207	38.4	
Sex							
Female	154	50.7	124	51.0	278	50.8	0.93
Male	150	49.3	119	48.9	269	49.2	
Delivery^a^							
Vaginal	150	49.5	111	45.8	261	47.8	0.42
Cesarean	153	50.5	132	54.3	285	52.2	
Gestational age							
<33	110	36.2	82	33.7	192	35.1	0.58
33-36	102	33.6	86	35.4	188	34.4	
≥37	92	30.3	75	30.9	167	30.5	
Birth weight (g)							
<1,000	18	5.9	18	7.4	36	6.6	0.005
1,000-1,499	51	16.8	17	6.9	68	12.4	
1,500-2,499	111	36.5	90	37.0	201	36.8	
≥2,500	124	40.8	118	48.6	242	44.2	
5-minute Apgar							
<7	37	12.2	26	10.7	63	11.5	0.68
≥7	267	87.8	217	89.3	484	88.5	
NICU admission							
Yes	197	64.8	185	76.1	382	69.8	0.005
No	107	35.2	58	23.9	165	30.2	

NICU: Neonatal Intensive Care Unit; ^a^Pearson’s chi-square test; α=5%; ^b^one case excluded in 2012: information ignored; ^c^seven cases excluded in 2012 and one in 2016: information ignored.

Table 2 shows that NICU admission was the entry criterion responsible for exclusively classifying the highest number of newborns as early neonatal near misses in both years, increasing from 36.2% in 2012 to 47.3% in 2016.

**Table 2:** Characterization of early neonatal near misses by entry criterion (exclusively by the criteria^a^). Hospital das Clínicas, 2012 and 2016.

Criteria	2012 n=304		2016 n=243		p-value^b^
	n	%	n	%	
NICU admission	110	36.2	115	47.32	0.008
Gestational age <33 weeks	37	12.2	31	12.76	0.834
Birth weight <1,750 g	28	9.2	8	3.29	0.005
5-minute Apgar <7	12	3.9	12	4.93	0.578

NICU: Neonatal Intensive Care Unit; ^a^cases classified as early neonatal near miss by only one criterion; ^b^Pearson’s test; α=5%.

We identified variations in neonatal near miss indicators and early neonatal mortality rate according to the studied year, with worse outcomes and more deaths in 2012, despite the higher early neonatal near miss rate (Table 3).

**Table 3:** Comparison of neonatal near miss indicators. Hospital das Clínicas, 2012 and 2016*.

Indicators	2012	2016	p-value^a^
Early neonatal near misses	304	243	--
Number of early neonatal deaths	33	25	--
Early neonatal deaths with risk conditions at birth	31	23	--
Early neonatal mortality rate^b^	15.74	10.19	0.131
Neonatal near miss rate^b^	144.97	99.02	<0.001
Severe neonatal outcome rate^b^	160.71	109.21	<0.001
Early neonatal survival index (%)	90.8	91.4	0.097
Early neonatal mortality index (%)	9.3	8.7	0.925

^a^Prevalence ratio; ^b^per thousand live births; *total number of live births: 2,097 in 2012 and 2,454 in 2016.

## DISCUSSION

Neonatal near miss indicators showed differences between the years analyzed, with a worse situation evidenced in 2012. In contrast, the number of NICU admissions was higher in 2016, demonstrating the usefulness of these markers in monitoring institutional neonatal care.

The limitations of this study are related to the use of secondary data, due to the possibility of under-registration, incompleteness, and inconsistency of SIM, Sinasc, and SIH-SUS data, which was reduced by the information retrieval performed by the hospital epidemiology center and the municipality’s Health Department. The coverage of vital information in Pernambuco is considered high,^16^ and the level of Sinasc and SIM implementation is adequate.^17^ The method used may not be appropriate to compare hospitals of different complexities or located in cities where the coverage, completeness, and reliability of information systems are insufficient without additional care.^5^ ^,^ ^11^ We overcame the problem by comparing the same hospital at different times.

The concept of neonatal near miss can be used as a severity grade, indicating near-death situations; however, it is conditioned by the definition chosen to identify cases. Sensitivity and specificity change depending on the adopted criteria, which will reflect on the number of newborns classified as surviving risk conditions at birth.^11^ ^,^ ^12^ The definition used in this study adopts the NICU admission criterion as a marker of case severity, allowing us to identify newborns who faced extreme situations that led to near death experiences.

Also, this definition is simple, data are easy to collect, and, if formulated based on variables obtained from good quality official information systems, its implementation as a neonatal care surveillance tool that can monitor and compare the performance of health care facilities over time becomes easier. Other existing definitions make data collection more complex, hindering its routine use in health services.^11^

Some studies suggest that the concept of neonatal near miss can assist in assessing the quality of hospital care for newborns.^7^ ^,^ ^18^ ^,^ ^19^ Nevertheless, the current definitions of neonatal near miss were constructed based on the epidemiological risk model related to early neonatal death. A thorough assessment of the quality of newborn care demands additional constructs from different perspectives (health professionals, management, users). The complexity of institutional evaluation processes calls for special attention regarding the profile of the users assisted, the health status severity of the population treated at the health facility, and the available and utilized medical technology.^20^ Comparing early neonatal near miss indicators or neonatal mortality rates of institutions with different profiles may lead to misinterpretations, requiring extra attention; however, this temporal comparison of the same health facility might serve as a preliminary warning of possible hospital care failures, complemented by the profile characterization of near misses.

The number of early neonatal near misses in twin pregnancies was higher in 2016. *Pesquisa Nascer no Brasil*, a national hospital-based study that analyzed data from 266 maternity hospitals, found a strong association of twin newborns with neonatal death (odds ratio between 5 and 7).^21^ In contrast, some studies do not confirm the association after multivariate analysis, probably because prematurity and low birth weight are quite prevalent among twins.^22^ ^,^ ^23^

The greater the number of prenatal visits, the higher the probability of receiving essential care to carry the pregnancy to term with desirable maternal and perinatal outcomes.^24^ Research conducted in public maternity hospitals in São Paulo and Rio de Janeiro evaluated factors related to neonatal near misses and deaths and identified failures in prenatal care in 80.8% of cases.^6^ In Northeastern Brazil, a study performed in a hospital qualified for high-risk pregnancy care revealed an association between less than six prenatal visits and an increase in the risk of neonatal near misses.^24^ In this study, a high proportion of mothers had zero or up to three prenatal visits in 2012, which corroborates the information that flaws persist in prenatal care, such as insufficient number of visits, assistance delay, and inadequate care, which have an impact on the morbidity and mortality of the mother-child dyad.^25^

Low birth weight is a known risk factor for early neonatal death in both population-based and hospital-based studies,^2^ ^,^ ^21^ ^,^ ^26^ even in cities with a low infant mortality rate,^23^ which is why this variable is used as a criterion to identify neonatal near misses.^9^ ^,^ ^11^ In this study, the proportion of early neonatal near misses with very low birth weight in 2012 was more than twice that of 2016.

In 2012, the incidence of early neonatal near misses with very low birth weight and mothers who had few or no prenatal visits was higher, while NICU admission predominated in 2016. These findings raise questions on the need for NICU referral and organizational problems that may have occurred in that year. The studied hospital complies with Ministerial Decree No. 930,^27^ which defines the guidelines and objectives for the organization of comprehensive and humanized care for newborns with severe or potentially severe conditions, with regard to the NICU admission criteria. The recommendation after NICU discharge is that the baby should stay in the conventional intermediate care unit or kangaroo, and later in the joint accommodation. In 2016, the institution investigated showed organizational problems related to the availability of beds due to the renovation of the intermediate care unit, among others, which may have overestimated the classification of early neonatal near miss, as babies who might not have needed hospitalization remained in the NICU because of inadequate referral or structural issues.

The concept of neonatal near miss, when explaining different situations, can highlight flaws in the management or organization of services that provide newborn care.^7^ ^,^ ^18^ It gives a warning but does not show the specificity of the problems to be faced, requiring further investigations, either by monitoring neonatal death or near misses, or by evaluating the service. The temporary deactivation of the intermediate care unit in 2016 affected the user profile, as the hospital studied started to admit only low-risk pregnant women. If newborns needed specific interventions, they were transferred to NICU, which influenced the identification of early neonatal near misses in that year and showed that such indicators were conditioned by the context of the place investigated, endorsing the claims that results are also related to the user profile.^20^

The incidence of early neonatal deaths and early neonatal near misses was higher in 2012 than in 2016. A greater number of near misses does not necessarily indicate a better result, and the ENMI or ENSI must be considered when analyzing the real proportion of deaths and survivals, respectively. In this study, despite the higher number of early neonatal near misses in 2012, the percentage of deaths in relation to newborns who had risk conditions at birth was higher and the percentage of survival was lower when compared to 2016. We cannot state that neonatal care was worse in 2012, particularly considering the change in user profile, and more in-depth evaluation studies are necessary to analyze the association between the concept of neonatal near miss and the quality of newborn care.

Our results indicate that early neonatal near miss indicators can monitor variations in morbidity and mortality in hospitals and maternity hospitals, allowing the identification of atypical situations that need detailed investigation in the service. Therefore, they can be used as a newborn health management and surveillance tool in tertiary health services.
